# Construction and Content Validation of Mobile Devices’ Application Messages about Food and Nutrition for DM2 Older Adults

**DOI:** 10.3390/nu16142306

**Published:** 2024-07-18

**Authors:** Rafaella Dusi, Raiza Rana de Souza Lima Trombini, Alayne Larissa Martins Pereira, Silvana Schwerz Funghetto, Verônica Cortez Ginani, Marina Morato Stival, Eduardo Yoshio Nakano, Renata Puppin Zandonadi

**Affiliations:** 1 University of Brasília, Faculty of Health Sciences, Department of Nutrition, Campus Universitario Darcy Ribeiro, Brasilia 70910-900, Brazil; vcginani@unb.br; 2 University of Brasília, Graduate Program in Health Sciences and Technologies, Campus Universitario Ceilândia, Brasília 72220-275, Brazil; raiza.lima@aluno.unb.br (R.R.d.S.L.T.); pereira.alayne@aluno.unb.br (A.L.M.P.); silvanasf@unb.br (S.S.F.); marinamorato@unb.br (M.M.S.); 3 University of Brasília, Department of Statistics, Campus Universitario Darcy Ribeiro, Brasilia 70910-900, Brazil; nakano@unb.br

**Keywords:** nutrition, type 2 diabetes mellitus, Brazilian older adults, mobile health interventions

## Abstract

Older adults face a decline in the quality of their diet, which affects their health. The prevalence of DM2 is increasing, as are the associated complications. Effective nutrition education and mobile health (mHealth) interventions offer a viable solution in the scenario of the widespread use of mobile devices. This study aimed to develop and validate messages for a mobile application aimed at older adult Brazilians with DM2 who receive care at the Brazilian Unified Health System (SUS). The educational messages on healthy eating for older adults with DM2 were created from 189 excerpts selected from Brazilian official documents. A total of 37 messages were created, categorized into 20 educational, 12 motivational, and 5 congratulatory, all up to 120 characters. Twenty-one experts validated the messages for clarity and relevance, and 11 messages had to be revised to meet the criteria. Subsequently, the 36 messages approved by the experts were tested on a sample of 57 older adults, guaranteeing clarity rates of over 80%. This study developed and validated 36 messages for a mobile health app aimed at older adults with type 2 diabetes mellitus in Brazil. Expert evaluation ensured clarity and relevance, confirmed by older adult participants who evaluated clarity. This research highlights the potential of mHealth to overcome barriers to accessing healthcare in the SUS, emphasizing personalized interventions for the effective management of older adults’ health.

## 1. Introduction

In Brazil, people aged 60 and over are considered older adults, and it is estimated that they will represent 18.6% of the population in 2030 and 33.7% in 2060 [1,2]. Older people tend to decline in dietary quality over the years, consuming a lower amount of total calories, proteins, and micronutrients than recommended but with an increased consumption of sodium and sugars. Such changes can be attributed to physiological changes in gastrointestinal absorption, the perception of hunger and satiety, oral health, and psychosocial aspects such as social isolation and financial insecurity [3].

Physiological changes, unhealthy eating patterns, a lack of physical activity, and other aspects related to aging make it closely linked to type 2 diabetes mellitus (DM2), and its prevalence increases with age [4]. DM2 is considered a global public health problem. Around 537 million older adults worldwide live with DM2 [5]. It has been estimated that the number of older adult patients with DM2 will quadruple worldwide in the next 3 decades [4]. In Brazil, it is estimated that one in four older adults presents DM2 (more than five million older adults) [6].

The older the Brazilian patients with DM2, the higher the rate of complications and hospitalizations. Older adults between 60 and 69 years old represent the largest portion of those hospitalized, while those over 70 represent the largest portion of deaths attributed to DM2 [7]. The most common implications associated with DM2 in older adults are lower limb injuries, ophthalmological and kidney diseases, strokes, and psychological disorders, such as depression [8] and mood changes [9]. In this sense, health promotion and prevention actions aimed at older adult patients with DM2 can be adopted to enable the individual to develop healthy habits that positively affect their quality of life [9]. Emotional suffering in older adults related to DM2 is generally linked to less adherence to nutrition-related self-care [10]. This reinforces the importance of food and nutrition education (FNE), which is effective and easy to follow [11].

Nutritional recommendations for patients with DM2 aim to assist in effectively controlling glycemia, weight gain, and systemic blood pressure so that their eating habits are mediators that reduce the risk of complications due to the decompensation of the disease [11]. In older adults, the intention to engage in a diet is eight times lower than in adults or young people [12]. Therefore, the nutritional strategies adopted in the care of DM2 should not only be prescriptive but also need to consider each patient’s stages of readiness for change and the behavioral aspects that determine food intake [11]. In this sense, diabetes education and food and nutrition education (FNE) must be adopted since diagnosis, focusing on the importance of self-care for managing the disease [11], potentially reducing morbidity and mortality in diabetic patients, and improving markers of glycemia, such as glycated hemoglobin (HbA1C), and decreasing complication rates [13]. Furthermore, older adults who clearly understand the relationship between food consumption and health outcomes are more likely to make conscious changes to their diet to ensure good health [12].

Strong evidence suggests that FNE actions, which are applied to individuals with DM2 for at least three months, can control HbA1C levels [14]. It is also noteworthy that interventions that focus on the psychosocial aspects of food and illness are more effective than those that only teach about nutrients and prescribe diets and that actions that are applied through mobile applications are also effective in reducing HbA1C levels in patients [14].

The Brazilian Unified Health System (SUS) faces enormous challenges, some related to the country’s territorial dimension and others to management. The insufficiency and difficulty of access to health professionals, the long distances between health reference centers and rural cities, and the lack of medication are some of the obstacles that older adults served by the SUS need to face. Considering these gaps, it is important to explore alternatives to offer health education and continuous, facilitated support for older adults with DM2 [15]. Despite the universality characteristic of the SUS, in 2019, 3.8% of the population reported unmet needs for health services, and the most common barriers are financial and geographic issues, the availability of resources [16], and long waiting times [17,18].

Mobile health (mHealth) is a medical and public health practice supported by mobile devices [19]. This innovative approach has shown potential in improving the treatment of older adult patients with chronic illnesses, facilitating treatment adherence, communication, understanding self-monitoring, and behavior change. Furthermore, mHealth supports health education, promoting great potential to effectively prevent and control diseases [19,20].

With the aging of the population and an increase in the number of older adults in Brazil, it is common to search for technological tools to ensure that this stage of life is spent in a healthy way and that individuals can remain active and independent [21,22]. In addition to the growing number of mobile device users, offering education about diabetes through these devices can be a strategic way to manage this health condition [23]. In this sense, health applications can be a workable solution to face some of these challenges, with technological innovation being applied to provide more efficiency in public health services [24].

Health interventions that use mobile technology provide a broad reach, allowing users to access information when they deem it most appropriate, wherever they are, and data show that older adults routinely adopt technology [19]. It is already known that older adults accept applications that send instant messages well, that text messages are useful in improving the health of DM2 patients, and that they can enhance the behavioral habits of patients with chronic diseases in less developed areas [25,26,27].

There is an important point to be observed when thinking about using these technologies for managing chronic diseases aimed at older adults: adaptation to the needs and abilities present in older adults, seeking to facilitate the use and adherence to the tool [24].

To develop mHealth responsibly, especially for older adults’ care and health education, it is essential to involve a multidisciplinary team. This team should include technology and healthcare experts and user representatives from the target audience. A crucial step in the initial phases of development is content validation, ensuring that the developed technology correctly meets healthcare aims when applied to the target audience and measuring exactly what it proposes [20]. To ensure that an instrument achieves its objective accurately, the validation of its content is crucial [28]. The quality of the instruments developed is crucial to the validity of the results of a study, which highlights the importance of the content validation process as an essential aspect [29].

Considering the potential for the prevention and control of DM2 that FNE actions have, the growing incidence of DM2 in the Brazilian population, the increase in life expectancy, and the promising scenario of research that uses mobile applications as a tool for health education, and the broad access of older adults to cell phones, this study aimed to construct and perform the content validation of application messages about food and nutrition for mobile devices for DM2 older people.

## 2. Materials and Methods

### 2.1. Study Design and Ethical Approval

This is a quantitative methodological study. This research is part of the project “Technologies for Older Adults Health Management and Self-Care”, approved by the Research Ethics Committees of the Faculty of Ceilândia of the University of Brasília—CEP/FCE (5.142.057 and CAAE: 51915021.9.0000.8093) and the Foundation for Teaching and Research in Health Sciences—CEP/FEPECS (5.237.938 and CAAE: 51915021.9.0000.8093).

### 2.2. Message Construction

Educational messages about healthy eating in the context of older adults with DM2 will form part of the food interface of an application aimed at managing DM2 in older adults from the SUS in Brazil. To prepare the content, a group of researchers evaluated the content of the following documents “*Alimentação Cardioprotetora: manual de orientações para profissionais de saúde da Atenção Básica*”—“Cardioprotective nutrition: guidelines for primary care health professionals” [30]; “*Guia Alimentar para a População Brasileira*”—“Dietary Guidelines for the Brazilian Population” [31]; and “*Orientação Alimentar de Pessoas Adultas com Diabetes Mellitus—Protocolo de Uso do Guia Alimentar para a População Brasileira—Volume 4*”—“Dietary Guidelines for Adults with Diabetes Mellitus—Protocol for the Use of the Dietary Guidelines for the Brazilian Population—Volume 4” [32].

The research group read the documents and selected excerpts from each one. These excerpts were then analyzed, and similar content was either merged or excluded. During the content evaluation, the excerpts were divided into four categories based on their content: basic guidelines, food groups, substitutions, and preparation and food shopping guidelines. This categorization was only used to facilitate the identification and removal or adjustment of duplicate or similar content. The complexity of the excerpts was assessed and classified into three levels: Low Complexity, Medium Complexity, and High Complexity. This classification was only used to help the research group determine whether any adaptations were needed to clarify the content for the study population.

Next, the messages were categorized based on their role in the mobile application: to motivate, educate, or congratulate, and their content was compared. Messages with similar content were further analyzed, and the team selected only one from each set to be included in the final document for expert evaluation.

### 2.3. Experts’ Message Validation (Clarity and Relevance)

Two PhDs and a master’s degree holder in nutrition evaluated the selected messages and made suggestions for refinement before experts assessed them. To ensure the messages fit on the mobile device screen as notifications, they were limited to 120 characters, including spaces.

For the first stage of validating the content of the messages about healthy eating in the context of older adults with DM2, 27 experts were invited (since Delphi methods require 10 to 18 experts minimum on a Delphi panel) [33], between November 2023 and February 2024. The experts were selected based on the method proposed by Fehring [34,35], with adaptations. The original Fehring criteria were aimed at selecting nurse experts, and in this study, we adapted them to include experts in nutrition, older adults’ health, and/or diabetes, thus maintaining the focus on this research’s object of study. The inclusion criteria for this group to participate in the study were as follows: being a health professional, achieving a score of ≥5 on the criteria adapted from Fehring (Table 1), agreeing to participate in the research, and responding to all forms relating to the research. Experts were excluded from the study if they did not submit their evaluation of educational technologies on time or if they participated only partially in the study. The messages were entered into Google Forms, and the experts responded on a five-point Likert scale to clarity (ranging from “not clear” to “totally clear”) and relevance (ranging from “not relevant” to “extremely relevant”), whether they agreed or disagreed with the clarity and whether they agreed or disagreed with the message’s relevance to the application. To be approved, the messages had to have an approval rating above 70% (with a mean Likert scale ≥ 4) [36].

Messages that did not reach 70% [36] agreement among the experts were sent for a new round of evaluation, after the adjustments they recommended [29]. The messages were entered into Google Forms, and the experts responded on a 5-point Likert scale (ranging from completely disagree to completely agree) whether they agreed or disagreed with the clarity of the message.

### 2.4. Target Audience Message Validation (Clarity)

The last step (pretesting) involved sending the messages approved for the experts to a convenience sample of older adults unfamiliar with the research. These individuals were asked to judge the clarity of the messages. The messages were placed in Google Forms, and the link was sent to the convenience sample. The participants responded using a 5-point Likert scale (ranging from “not clear” to “totally clear”) to indicate whether they found the messages clear. To be approved, the messages had to have an approval rating above 80% (with a mean Likert scale ≥ 4) [37].

### 2.5. Statistical Analysis

The obtained data were extracted from Google Forms© into a Microsoft Excel© version 2406 spreadsheet, and the software IBM SPSS© (Statistical Product and Service Solutions), version 22, was used to analyze the data. The characteristics of the sample subjects were categorized and presented by frequency and percentages.

## 3. Results

**Figure 1:**
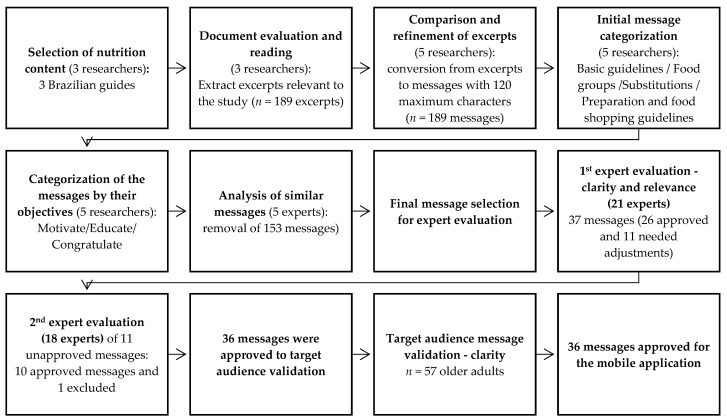
A flowchart of this study’s steps.

This study was conducted in three stages: the construction of the messages, experts’ evaluation (relevance and clarity), and target audience evaluation (clarity). Figure 1 summarizes the steps of this study, and then all results are explained in each step.

### 3.1. Message Construction

A total of 189 excerpts were selected from the three Brazilian government documents [30,31,32]. After analyzing the content and similarities between the texts, excluding duplicates, and organizing the excerpts into messages, a total of 37 messages were created about healthy eating in the context of older adults with DM2, categorized as 20 educational, 12 motivational, and 5 congratulatory. All of the messages had up to 120 characters.

The educational messages (*n* = 20; 54.1%) provide educational guidance on possible substitutions for unhealthy foods, the harms of ultra-processed foods, the impact of food and alcohol on blood sugar, and how to manage mealtimes. The motivational ones (*n* = 12; 32.4%) focus on the results of eating on health and physical and social well-being. Finally, the congratulatory messages (*n* = 5; 13.5%) recognize the learning and effort made to improve their eating routine while using the app. The researchers discussed all the messages, and afterward, they were sent to the experts for evaluation and validation.

### 3.2. Experts’ Message Validation (Clarity and Relevance)

A total of 27 experts who met the inclusion criteria were invited to participate in this stage to achieve the minimum sample size described in the Delphi method [33]. Of these, 21 experts accepted the invitation to participate in the message validation process. Approximately 91% (*n* = 19) had degrees in human nutrition, 81% (*n* = 17) were female, and 81% (*n* = 17) were between the ages of 31 and 54. Furthermore, 85% (*n* = 18) held a master’s degree or PhD in health, 33.3% (*n* = 6) had published articles on older adults with diabetes mellitus/mHealth/primary healthcare, and 80.9% (*n* = 17) had published articles on health education/validation studies. Additionally, 57.1% (*n* = 12) were engaged in teaching, and 23.8% (*n* = 5) were involved in research (see Supplementary File—Table S1).

The experts evaluated the messages for clarity and relevance between November 2023 and March 2024. Out of 37 messages, 11 did not reach 70% approval (with a mean Likert scale ≥ 4) [36] and were sent to the second round of experts’ evaluation. The experts’ comments on these 11 messages were assessed, and the texts were adjusted by the research group to be resubmitted for clarity reassessment.

The 11 revised messages (9 educational, 1 motivational, and 1 congratulatory) were reinserted into a Google form, and the same 21 experts received the link to reassess their clarity in May 2024. A total of 18 of the 21 experts agreed to re-evaluate, and at the end of this stage, 10 messages were approved, with over 70% agreement for clarity [36]. Only one educational message was excluded, as it did not achieve 70% clarity agreement. By the end of the validation process, all 36 messages were approved for target audience validation. Approved messages in Brazilian Portuguese and their English translations are shown in Table 2. The English translations are included only to facilitate the reader’s understanding of the study.

### 3.3. Target Audience Message Validation (Clarity)

At the final stage, the 36 messages approved by the experts were analyzed in June 2024. The messages were sent to 65 people aged ≥60, aiming to achieve a minimum of 50 participants (10 participants for each of the five possible answers). Of these, 60 agreed to participate, but only 57 accepted the consent terms and completed the evaluation. The participants in this stage were predominantly female (56.1%; *n* = 32), with an average age of 68.2 ± 8.3 years old, mostly holding postgraduate degrees (47.3%; *n* = 27), and with an income exceeding nine minimum wages (approximately USD 2400) (66.6%; *n* = 38) (Supplementary File—Table S2).

Of the 36 messages, all of them had over 80% (with a mean Likert scale ≥ 4) in the first round [37] and were finally approved to be part of the app.

## 4. Discussion

This study focused on constructing and validating the content of food and nutrition messages for mobile apps designed for older adults with DM2. The study design was crucial to ensure that the messages included in the app passed all the necessary approval stages. Validating the content with both experts and the target population is essential for this purpose [38].

Facilitating treatment adherence, communication, understanding self-monitoring, and behavior change, mHealth supports health education and shows great potential to effectively prevent and control diseases [19,20]. Additionally, interventions that address the psychosocial aspects of food and deliver actions through mobile applications effectively improve DM2 control in patients [14].

It is also known that older adults served by the SUS in Brazil face obstacles such as difficulty accessing health professionals [15]. This reinforces the importance of the app’s messages, which can potentially reduce these barriers and offer alternatives for self-care for older adults with DM2.

### 4.1. Message Construction

In our study, three dietitian experts critically analyzed the content excerpts from the text. They were extensively familiar with the documents to ensure the selection of essential messages that fulfill the intended function in the mobile app. In another study aiming to improve HbA1C levels and diabetes self-management activities, the authors highlighted the importance of nutrition experts reviewing and editing the content to ensure validity and cultural appropriateness [39]. The construction of the messages by a panel of experts is also cited as an essential step in another study that looked at the role of text messaging intervention in Inner Mongolia among patients with DM2 [27].

Extracting the messages from previously validated documents published by official Brazilian government bodies guarantees that experts in the field of nutrition have already evaluated the base content. These documents are fundamental guides for the nutrition of the Brazilian population, which have undergone a rigorous review by a technical body before publication [30,31,40]. Therefore, this information was converted into shorter messages (up to 120 characters) that are easy to access and read.

### 4.2. Experts’ Message Validation (Clarity and Relevance)

The quality of the instruments developed is key to ensuring the validity of study results, underscoring the critical role of the content validation process as an indispensable factor [29].

Validating the content of an instrument is essential to ensure it achieves its objectives accurately [28]. Content validation ensures that the developed technology effectively meets healthcare goals when implemented among the target audience, accurately measuring its intended outcomes [20]. Content validation by experts in nutrition is considered crucial because it ensures that individuals with technical knowledge evaluate quality and relevance, which is vital in developing instruments to ensure their effectiveness [25,41]. This is consistent with our expert panel, in which more than 90% of them had degrees in human nutrition.

During the validation phase, experts must have knowledge of the research topic to accurately assess the relevance and clarity of the content under review [35]. In our study, it was crucial that messages initially not approved were revised based on expert recommendations and re-evaluated. Subsequently, 10 out of the initial 11 messages that were reformulated according to the experts’ suggestions were approved for definitive use with the target population, along with the rest of the others, totaling 36 approved messages overall.

### 4.3. Target Audience Message Validation (Clarity)

The participants’ high education and income levels are likely due to the online nature of this stage, which attracted a convenient sample. This is a potential limitation of our study. Regions with greater development typically enjoy better internet access, and internet users often possess higher education and income levels. Those with advanced education are more inclined to utilize online resources, and higher income levels significantly influence participation in health surveys [42,43,44,45]. Despite the prevalence of high education and income levels, our sample presented participants with low levels of education (7.1%) and income (33.2%) who also approved the messages regarding clarity.

Studies involving diabetic women (mean age 52 years) have shown that patients with type 2 diabetes appreciate reminders via text messages. Additionally, research with adults (more than 69% over 50 years) diagnosed with type 2 diabetes has demonstrated that text messages effectively promote the adoption of healthy behaviors, offering an economical and practical intervention [27,46].

All of the messages had over 80% approval (with a mean Likert scale ≥ 4) [37] to be included in the app. Mobile health interventions provide widespread access to information and are increasingly favored by older adults who are embracing technology [19]. Adapting technology to manage chronic diseases in older adults is crucial for improving usability and adherence to the tools, considering their specific needs and capabilities [24]. This highlights the importance of assessing the clarity of the messages that will be part of the app with older adults, ensuring that it resonates effectively with the target audience.

## 5. Limitations

Despite the methodological rigor applied in this study, limitations should be highlighted. There may be selection bias, as we used a convenience sample likely composed of older adults more familiar with technology, necessary for participating in an online stage. There is also potential bias with the experts due to their prior familiarity with the research documents, which are widely disseminated in Brazil. This familiarity might have influenced their evaluations.

The target audience evaluation reached a small population characterized by high income and education levels. This may not have encompassed a variety of people who might have different perspectives, such as regional dialects, literacy levels, and cultural beliefs about health and nutrition, which could affect how messages are received and understood. Addressing these limitations in future research could enhance the robustness and applicability of the findings, ensuring that the developed mobile health interventions are more inclusive, culturally sensitive, and effective in supporting older adults with DM2.

## 6. Conclusions

This study developed and validated a series of 36 messages designed to be part of a mobile health app in the future aimed at improving eating habits among older adults diagnosed with type 2 diabetes mellitus (DM2) in Brazil. Expert evaluation ensured the clarity and relevance of these messages, which were then confirmed by older adults themselves to ensure their comprehension and applicability in the app.

The results highlight the potential of mobile health technologies to overcome barriers to accessing healthcare, especially in the structure of Brazil’s SUS, where challenges persist, such as limited access to healthcare professionals and resources. In summary, this research offers valuable insights into developing and validating health communication strategies through mobile apps, highlighting the importance of personalized interventions to empower older adults to manage their health effectively.

## Figures and Tables

**Table 1:** Scoring system and criteria adapted from Fehring for selecting experts.

Criteria	Score
Holds a master’s degree/PhD in the health field	3 points
Holds a master’s degree with a dissertation on older adults with DM2/mHealth/primary healthcare	2 points
Holds a PhD with a thesis in the area of older adults with DM2/mHealth/primary healthcare	2 points
Has a published article on older adults with DM2/mHealth/primary healthcare	2 points
Published articles on health education/validation studies	1 point
Has a recent clinical practice of at least one year in primary healthcare	2 points
Has training (specialization) in older adults with DM2/mHealth/primary healthcare	2 points
Total	14 points

**Table 2:** Approved messages in Brazilian Portuguese and free translation into English.

Messages in Brazilian Portuguese	Messages in English *
*Sua alimentação deve ser baseada em alimentos naturais, como: frutas, hortaliças (verduras e legumes), cereais integrais (arroz integral, aveia etc.) e leguminosas (feijão, grão-de-bico etc.).*	Your diet should be based on natural foods, such as fruit, vegetables, whole grains (brown rice, oats, etc.), and legumes (beans, chickpeas, etc.).
*O consumo de doces e bebidas açucaradas (refrigerantes, refrescos em pó, sucos com açúcar) pode levar ao aumento de peso e de açúcar e gordura no sangue.*	The consumption of sweets and sugary drinks (soft drinks, powdered drinks, juices with sugar) can lead to weight gain and an increase in sugar and fat in the blood.
*Nem todo alimento “diet”, “light” ou “fit” é indicado para pessoas com diabetes, pois pode conter alta quantidade de gordura, sal, açúcar ou adoçante. Leia os rótulos.*	Not all “diet”, “light”, or “fit” foods are suitable for people with diabetes, as they may contain a high amount of fat, salt, sugar, or sweetener. Read the labels.
*Consumir hortaliças (verduras e legumes), frutas, leguminosas (feijão, lentilha etc.), carnes, ovos e leite favorece o controle do açúcar no sangue e da fome.*	Eating vegetables, fruit, legumes (beans, lentils, etc.), meat, eggs, and milk helps control blood sugar and hunger.
*Consumir alimentos integrais como arroz, pão, macarrão, faz parte da alimentação saudável, especialmente, para pessoas com diabetes.*	Eating whole-grain foods such as rice, bread, and pasta is part of a healthy diet, especially for people with diabetes.
*Aves, peixes e ovos são ricos em proteínas e emvitaminas, além de serem bons substitutos para as carnes vermelhas.*	Chicken, fish, and eggs are rich in protein and vitamins and good red meat substitutes.
*Bebidas alcoólicas podem atrapalhar o controle do açúcar no sangue, favorecer o ganho de peso e o aumento da pressão arterial.*	Alcoholic drinks can hinder blood sugar control, encourage weight gain, and increase blood pressure.
*Evite alimentos industrializados como salsicha, salgadinhos de pacote e refrigerantes, pois não são bons para a Saúde.*	Avoid industrialized foods such as sausage, packet snacks, and soft drinks, as they are not good for your health.
*Nas refeições ou lanches, coma primeiro as saladas e frutas, elas ajudam você a ficar mais satisfeito e será melhor para sua saúde.*	At mealtimes or snacks, eat salads and fruit first; they will help you feel more satisfied and will be better for your health.
*Combinar o iogurte natural com frutas e aveia ou sementes (linhaça, gergelim etc.) é uma boa opção para um lanche saboroso e saudável.*	Combining natural yogurt with fruit and oats or seeds (linseed, sesame, etc.) is a good option for a tasty and healthy snack.
*Congelar alimentos feitos em casa (como arroz, feijões, carnes e vegetais) e consumir durante a semana ou mês, facilita bastante a organização da sua alimentação.*	Freezing homemade food (such as rice, beans, meat, and vegetables) and consuming it during the week or month makes it much easier to organize your diet.
*Distribuir as refeições ao longo do dia (café da manhã, lanche, almoço, jantar e ceia) ajuda a comer quantidades de alimentos suficientes, evitando a fome extrema.*	Distributing your meals throughout the day (breakfast, snack, lunch, dinner, and supper) helps you eat enough food to avoid extreme hunger.
*A compra semanal de frutas e hortaliças (legumes e verduras) da estação fará que você tenha sempre alimentos saudáveis e mais baratos.*	Buying seasonal fruit and vegetables weekly will ensure that you always have healthy and cheaper food.
*Você deve cuidar da sua alimentação para ter mais qualidade de vida.*	You need to take care of your diet for a better quality of life.
*Organize a sua alimentação. Cuide de você!*	Organize your diet. Take care of yourself!
*Quando você adota uma alimentação saudável, inspira outras pessoas!*	When you eat healthily, you inspire others!
*Para a sua família e amigos é muito importante que você esteja bem! Portanto, cuide de sua alimentação.*	It is essential to your family and friends that you look good! So, take care of your diet.
*Alimentos naturais são mais saudáveis. Portanto, descasque mais e desembale menos!*	Natural food is healthier than industrialized food. So, peel more and unpack less!
*Leve com você alimentos saudáveis e que sejam fáceis de transportar e consumir, como frutas ou castanhas sem sal.*	Bring healthy foods that are easy to transport and eat, such as fruit or unsalted nuts.
*É possível preparar em casa comidas saudáveis. O nosso aplicativo tem algumas receitas. Prepare e compartilhe com quem você ama.*	You can prepare healthy food at home. Our app has some recipes. Prepare them and share them with your loved ones.
*Toda refeição saudável é um passo que você dá para uma vida melhor.*	Every healthy meal is a step towards a better life.
*Sua saúde é a principal motivação para comer refeições saudáveis! Nunca deixe de se cuidar.*	Your health is the main motivation for eating healthy meals! Never stop taking care of yourself.
*Cuidar da alimentação é muito bom! Sinta orgulho de você por escolher refeições saudáveis!*	Taking care of your diet feels good! Be proud of yourself for choosing healthy meals!
*Utilize receitas fáceis e saudáveis para facilitar o planejamento de sua alimentação*	Use easy and healthy recipes to make your meal planning easier.
*Todo dia é dia de decidir ser saudável. Parabéns pelo seu trabalho até aqui!*	Every day is a day to decide to be healthy. Congratulations on your work so far!
*Nesse período, você aprendeu muitas coisas sobre alimentação. Será cada vez mais fácil para você controlar o açúcar no sangue.*	During this time, you have learned a lot about food. It will become easier and easier for you to control your blood sugar.
*Conquistar uma alimentação equilibrada te ajuda a se sentir melhor em relação ao corpo e à saúde. Você também pode ser parte disso!*	A balanced diet helps you feel better about your body and health. You can be part of it too!
*Quando decidir consumir sobremesa, prefira frutas.*	When you decide to have dessert, go for fruit.
*Você pode substituir a linguiça, a mortadela e a salsicha por carne de boi, porco, frango, peixe ou queijos e ovo.*	You can substitute beef, pork, chicken, fish, cheese, and eggs for sausages, bologna, and sausages.
*Ter uma alimentação saudável pode ser desafiador, mas é um processo. Cada passo tornará esse caminho mais fácil!*	Eating healthy can be challenging, but it is a process. Each step will make it easier!
*Substitua os temperos industrializados prontos (tablete ou pó) pelos naturais, como: alho, cebola, alecrim, cebolinha, coentro, salsa, manjericão e limão.*	Replace ready-made industrialized spices (tablets or powder) with natural ones, such as garlic, onion, rosemary, chives, coriander, parsley, basil, and lemon.
*Comer sem pressa, com atenção e de forma saudável ajuda na digestão e no consumo adequado dos alimentos.*	Eating without haste, with attention, and in a healthy way helps digestion and proper food consumption.
*As frutas podem ser consumidas frescas, cozidas, assadas ou secas (desidratadas). Você pode comer a fruta pura em lanches ou incluir em saladas e sobremesas*	Fruit can be eaten fresh, cooked, baked, or dried (dehydrated). You can eat fruit as a snack or include it in salads and desserts.
*Fazer compras sem estar com fome favorece a compra de alimentos mais saudáveis e ajuda a economizar*	Shopping when you are not hungry helps you buy healthier foods and save money.
*Evite molhos prontos para saladas. Prefira opções mais saudáveis, como azeite, limão e vinagres ou molhos caseiros a base de iogurte natural*	Avoid ready-made salad dressings. Prefer healthier options such as olive oil, lemon, vinegar, or homemade sauces based on natural yogurt.
*Obrigada por aceitar participar desse programa! Você é essencial para que possamos ajudar outras pessoas.*	Thank you for agreeing to take part in this program! You are essential for us to be able to help other people.

* These messages were not validated in English. The free translation to English was performed to allow people of other cultures to understand the messages. The text in italics refers to the Brazilian Portuguese messages.

## Data Availability

The raw data supporting the conclusions of this article will be made available by the authors on request.

## Supplementary Materials

The following supporting information can be downloaded at https://www.mdpi.com/article/10.3390/nu16142306/s1, Table S1. Characteristics of the experts (*n* = 21 experts); Table S2. Characteristics of the individuals (*n* = 57 older adults).
