# Supplementary material for: Construction and Content Validation of Mobile Devices’ Application Messages about Food and Nutrition for DM2 Older Adults
Source: Nutrients. 2024 Jul 18;16(14):2306. doi: 10.3390/nu16142306 (PMC11280354; doi:10.3390/nu16142306)
Supplement: Supplementary file 1 [file nutrients-16-02306-s001.zip › nutrients-3092658-supplementary.pdf]

Table S1. Characteristics of the experts (n = 21 experts).

		Frequency *n = 21*	%
**Experts' gender**	Female	17	80.9 %
	Male	4	19.1 %
**Experts' age**	25 to 30 years	2	9.5 %
	31 to 39 years	8	38.1 %
	40 to 45 years	2	9.5 %
	46 to 54 years	7	33.4 %
	55 years and older	2	9.5 %
**Field of study**	Nutrition	19	90.5 %
	Others	2	9.5 %
**Master's degree or PhD in the health**	Yes	18	85.7 %
	No	3	14.3 %
**Holds a master's degree with a dissertation on older adults with DM2[1] / mH[2] / PHC[3]**	Yes	2	9.5 %
	No	19	90.5 %
**Has published an article on older adults with DM2[1] / mH[2] / PHC[3]**	Yes	7	33.3 %
	No	14	66.7 %
**Has published articles on health education/validation studies**	Yes	17	80.9 %
	No	4	19.1 %
**Has a recent clinical practice of at least one year in PHC[3]**	Yes	6	71.4%
	No	15	28.6 %
**Specialization in older adults with DM2[1] / mH[2] / PHC[3]**	Yes	5	23.8 %
	No	16	76.2 %
**Professional activity**	Teaching	12	57.1 %
	Research	5	23.8 %
	Health care	4	19.1 %

[1] Type 2 Diabetes Mellitus; [2] mHealth; [3] Primary Health Care

Table S2. Characteristics of the individuals (n = 57 older adults).

		Frequency *sample = 57*	%
**Older adults' gender**	Female	32	56.2 %
	Male	25	43.8 %
**Older adults' age**	60 to 69 years	37	64.9 %
	70 to 79 years	13	22.8 %
	80 to 89 years	6	10.5 %
	90 years and older	1	1.8 %
**Educational level**	Middle School	3	5.3 %
	High School	1	1.8 %
	Undergraduate	26	45.6 %
	Graduate	27	47.3%
**Monthly income** [1]	Up to 3 MW	7	12.2 %
	3 to 6 MW	5	8.8 %
	6 to 9 MW	7	12.2 %
	9 to 12 MW	11	19.3 %
	12 to 15 MW	12	21.2 %
	15 MW and more	15	26.3 %

[1] BRL: Brazilian Real is the official currency of Brazil and 1.00 USD = 5.38 BRL. Official minimum wage (MW) of BRL 1,412.00. (June 15, 2024).
